# Creative Arts to Enhance Cervical Cancer Awareness Using Art-Based Messages From a Nigerian Crowdsourcing Open Call: Qualitative Thematic Analysis

**DOI:** 10.2196/76240

**Published:** 2026-01-23

**Authors:** Abdulhammed O Babatunde, Ekenechukwu Kokelu, Olufunto A Olusanya, Peter Kalulu, Agatha E Wapmuk, Titilola Gbaja-biamila, Temitope Ojo, Ucheoma Nwaozuru, Chisom Obi-Jeff, Onyekachukwu Anikamadu, Folahanmi T Akinsolu, Hong Xian, Jennifer S Smith, Kayode O Ajenifuja, Jason J Ong, Benedict N Azuogu, Collins O Airhihenbuwa, Joseph D Tucker, Oliver C Ezechi, Juliet Iwelunmor

**Affiliations:** 1 College of Medicine University of Ibadan Ibadan Nigeria; 2 Clinical Sciences Department Nigerian Institute of Medical Research Lagos Nigeria; 3 Division of Infectious Diseases, John T Milliken Department of Medicine School of Medicine Washington University in St Louis St. Louis, MO United States; 4 Department of Implementation Science School of Medicine Wake Forest University Winston-Salem, NC United States; 5 Brooks Insights Abuja Nigeria; 6 Brown School of Social Work Washington University in St Louis St. Louis, MO United States; 7 College for Public Health and Social Justice Saint Louis University St. Louis, MO United States; 8 Department of Epidemiology University of North Carolina Chapel Hill, NC United States; 9 Obafemi Awolowo University Osun Nigeria; 10 Faculty of Infectious and Tropical Diseases Department of Clinical Research London School of Hygiene & Tropical Medicine London United Kingdom; 11 School of Translational Medicine Monash University Clayton Australia; 12 Melbourne Sexual Health Center Alfred Health Melbourne Australia; 13 Department of Community Health, Abakaliki Ebonyi State University Ebonyi Nigeria; 14 School of Public Health Georgia State University Atlanta, GA United States; 15 Institute for Global Health and Infectious Diseases School of Medicine University of North Carolina at Chapel Hill Chapel Hill, NC United States; 16 Department of Clinical Research Faculty of Infectious and Tropical Diseases London School of Hygiene & Tropical Medicine London United Kingdom

**Keywords:** art, cancer prevention, cervical cancer, education, HPV vaccines, social media

## Abstract

**Background:**

Cervical cancer is a leading cause of cancer deaths among women in Nigeria, yet awareness is low. Historically, art has served as a medium for processing emotions and sharing experiences, which can be effective in promoting health and behavior change.

**Objective:**

This study aimed to examine art submissions and social media engagement from a Nigerian crowdsourcing open call to inform co-designed strategies for cervical cancer prevention among women.

**Methods:**

This study reported following the SRQR (Standard Reporting for Qualitative Research) guideline. From October to November 2023, we launched an open call for art on social media, inviting adult women to submit artwork that raises awareness about cervical cancer prevention. Participants’ submissions were anonymized and reviewed by an independent panel of judges. A total of 6 finalists were selected to participate in a social media contest during Cervical Cancer Elimination Week, and 3 winners were identified based on total social media likes and comments, as well as grading rubric scores. We analyzed participants’ art entries through thematic analysis in six steps: (1) familiarization, (2) creating categories, (3) identifying themes, (4) reviewing themes, (5) defining themes, and (6) discussing findings. The emerging themes included using art to express emotions, to convey health-related content, and to use art as a form of self-regulation, which were further analyzed using the Relationships and Expectations domain of the PEN-3 (perceptions, enablers, and nurturers) cultural model.

**Results:**

A total of 43 entries from participants aged 18-27 (mean 22.2, SD 2.6) years were analyzed. The entries included visuals (n=14), audiovisual (n=14), text (n=14), and audio (n=1). Most entries (42/43, 97.6%) focused on cervical cancer and human papillomavirus, covering definitions, risk factors, treatment, and prevention. Using the PEN-3 cultural model’s Relationships and Expectations domain for analysis, 62.8% (27/43) addressed “perceptions” of art as a means of mental and emotional expression, reflecting feelings such as humor, sadness, hope, faith, unity, and fear related to cervical cancer prevention. A majority (34/43, 79.1%) aimed to promote human papillomavirus screening and vaccination and were categorized as “enablers,” viewing art as a tool for health awareness, including educational resources. Additionally, 7% (3/43) included “nurturers,” representing self-regulation addressing stresses linked to having relatives with cervical cancer. Six finalist entries were shared on social media to promote cervical cancer awareness, reaching 8685 individual Instagram accounts and generating 2727 likes and 782 comments.

**Conclusions:**

This study used art to increase awareness about cervical cancer on social media. Art can serve as a tool for promoting health by incorporating visual, emotional, and contextual messages to influence the target audience’s behavior.

## Introduction

Invasive cervical cancer is a leading cause of death among women, resulting in about 350,000 deaths yearly [1]. The burden of cervical cancer is highest in low- and middle-income countries [2], with the majority of the cases and deaths occurring in sub-Saharan Africa, Central America, and Southeast Asia [1]. Regional disparities in cervical cancer incidence are linked to unequal access to vaccination, screening, and treatment services, as well as risk factors that include HIV prevalence, social barriers, gender biases, and poverty [2]. In Nigeria, cervical cancer is the second leading cause of female cancer mortality in women aged 15-44 years [3]. About 12,000 cases and 8000 deaths are reported annually, with an incidence rate of 18.4 per 100,000 women [4]. Sociodemographic characteristics, including low levels of education, limited knowledge, and low levels of health-seeking behavior, are linked with a higher incidence and mortality rate of cervical cancer in Nigeria [5]. These issues arise from misconceptions, stigma, and fear, resulting in delays in seeking screening and care that lead to late presentation and worsened outcomes [5].

Cervical cancer is largely attributed to persistent high-risk human papillomavirus (HPV) infection, which is linked to about 99% of cervical cancer cases in women [6]. Thirteen types of high-risk HPV cause cervical cancer, with types 16 and 18 being the most common and responsible for approximately 70% of cervical cancer cases. HPV infection is the most common sexually transmitted infection, with the peak age of infection being 25 years [7]. The average age for global cervical cancer diagnosis is 59 years [8]. Studies have demonstrated that HPV vaccination is highly effective in preventing cervical cancer, particularly cancers caused by vaccine-associated HPV types 16, 18, 31, 33, 52, 58, 72, and 78 [6,9].

In 2020, the World Health Assembly launched a global initiative aimed at eliminating cervical cancer by 2030. The goal is to achieve an incidence rate of fewer than 4 cases per 100,000 people globally, alongside the 90-70-90 targets: vaccinating 90% of girls by age 15, screening 70% of women at least twice by 35 and 45 years, and treating 90% of women diagnosed with cervical cancer premalignant lesions [10]. This approach emphasizes the importance of HPV vaccination and screening in the fight against cervical cancer [11]. However, in some parts of Nigeria, there is a lack of awareness and knowledge about HPV vaccination and cervical cancer. A study in Lagos found that just about 12% of female participants aged 15-49 years were aware of cervical cancer, its risk factors, and modalities of prevention [12].

Additionally, research among women of reproductive age in Kwara State, Nigeria, found that only 39% were well-informed, and just 6.9% had undergone screening [13]. Similarly, in a study conducted in Taraba, Nigeria, among 978 women of reproductive age, religion, especially those who identified as Muslims, were less likely to know about cervical cancer and the practices of cervical cancer screening [14]. Adequate knowledge and awareness of HPV vaccination and cervical cancer screening have been shown to result in a higher uptake of these preventive services [15]. Furthermore, cultural values and beliefs have been shown to influence the use of health services among women in Nigeria [16]. Culture shapes beliefs, values, norms, and behaviors, influencing the type of health information an individual encounters while seeking health services.

Historically, art has been used to process emotions and convey experiences that can promote health, increase health awareness and advocacy, and potentially change behavior [17]. Bunn et al [18] reported that art-based approaches have been used in sub-Saharan African countries to raise awareness of infectious diseases such as HIV/AIDS, malaria, and Ebola. However, the potential of using art as a method to address cervical cancer morbidities and mortalities has not been fully explored. In response, we organized an art contest to increase awareness of cervical cancer in Nigeria, using a participatory action research framework, especially a crowdsourcing open call aimed at soliciting artwork among adult women to raise awareness and knowledge and ultimately reduce cervical cancer mortalities and morbidities. A crowdsourcing open call involves inviting a group of people, such as adult women impacted by cervical cancer, to solve a problem and share selected solutions with the public [19]. This research examines how art uses cultural contexts to enhance health education and promotion, specifically regarding perceptions of HPV vaccination and screening in Nigeria. We analyzed textual data from participants’ art submissions and social media interactions collected during an open art contest aimed at raising cervical cancer awareness among Nigerian women.

## Methods

### Study Design and Recruitment

Our approach is guided by a participatory action research framework that prioritizes integrating participation and action to understand and address societal issues, such as cervical cancer incidence and mortality [20,21]. We adhered to the SRQR (Standard Reporting for Qualitative Research) guideline [22].

### Crowdsourcing Open Call

An open call for artwork to promote cervical cancer awareness was launched on social media between October 3, 2023, and November 5, 2023. The artwork submitted included drawings, video art, animation, illustrations, poetry, drama, and more. We developed a range of promotional materials to provide information about the art contest, its benefits, and application links. These materials were shared on the 4 Girls and Women social media platforms (Instagram, X, LinkedIn, and WhatsApp) and distributed through health influencers. We also disseminated the open call through networks of girls and women who participated in the HPV Designathon in Nigeria. An informational webinar was hosted to guide interested applicants.

### Study Setting and Participants

This study includes women aged 18-65 years residing in Nigeria who submitted artwork in response to the open call. This was a purposive sampling approach targeting artists and creatives in Nigeria.

### Crowdsourcing Open Call Submissions

Submissions were made through Google Forms, email, or WhatsApp. We collected a range of sociodemographic data points, such as name, age, and sex, and submission-specific information, including the submission title and a brief synopsis. At the end of the open call, the entries received were anonymized and screened for eligibility by an independent panel of judges. These data elements helped to categorize participants’ submissions based on both content and demographic details.

### Entry Selections

Judges reviewed and evaluated 43 eligible entries using a grading rubric that included the level of innovation demonstrated in the submission, the clarity and effectiveness of expression, the originality and creativity of the content, the overall appeal and engagement factor of the submission, and its relevance and significance to public health. Each criterion was graded on a point scale from 0 to 5. Five judges evaluated each submission, and an overall score was recorded. Following this evaluation process, we shortlisted the entries that ranked in the top 10. To ensure the accuracy and reliability of the health-related information presented in these submissions, we then shared them with a panel of public health experts for further review [23]. Following this review of the top 10 entries, 6 finalists were shortlisted.

### Social Media Contest

From December 15 to 17, 2023, six finalists were shortlisted for the second phase of the social media contest. The contest was conducted on Instagram, Nigeria’s third most-used social media platform, following WhatsApp and Facebook [24]. Instagram is also the preferred social media platform for sharing images and videos. Its robust algorithms effectively promote content to target audiences through features such as hashtags [25]. The artwork from each of the 6 finalists was posted on the 4 Girls and Women account, in collaboration with the participants’ accounts, with a synopsis as the caption. The network among participants promoted the posts to drive engagement (views, likes, and comments) while educating users about cervical cancer. Three winners were determined based on their level of engagement on social media and the judge’s overall score, using the same judging rubric as in the first round. The winners were announced during an Instagram Live session on December 17, 2023, and were awarded cash prizes of NGN 200,000 (US $200), NGN 150,000 (US $150), and NGN 100,000 (US $100) for first, second, and third place, respectively. The 3 winning artworks are provided in references [26-28] and will also be featured in an international art magazine on health promotion.

### Data Analysis

A descriptive analysis examined the participants’ characteristics, including age, gender, ethnicity, and educational level. Additionally, the level of engagement with participants’ social media posts (eg, likes, shares, and comments) was analyzed, yielding valuable insights into digital reach and interactions.

For qualitative analysis, we adopted an inductive-deductive approach, categorizing submitted artwork into text-based, visual, audiovisual, and audio types. Following Lakh et al [29], our study used thematic analysis to identify emerging themes in the art submissions and to explore the underlying experiences and perspectives. Direct observation and interpretation of the submissions were also used to draw conclusions. Two researchers independently reviewed the submissions and resolved discrepancies; a third researcher was involved when discrepancies persisted, including an assessment of the credibility and accuracy of the two researchers’ reviews. For the thematic analysis, we used the 6-step process [30], which included (1) researchers’ familiarization with the submitted arts and accompanying texts, audios, or visuals, including identification of relevant items to the research question; (2) creation of categories based on the identified relevant items and comparing them back to the art entries; (3) identification of emerging themes, while comparing the art entries and the categories; (4) review of emerged themes while comparing the themes with the art entries, and the texts, audios, or visuals to ensure they were relevant to the research question, including refining, splitting, combining, and discarding some themes during this step; (5) naming and giving definition to the emerged themes, including organizing the themes; and (6) writing the findings, and discussion, while referring to the literature. The entire process involved referring to the initial art entries [29]. By following the 6 steps, themes emerged that outlined the key areas where art intersects with medicine and classified participants’ responses into the following three emerging themes: (1) art as an avenue for mental and emotional expression, (2) art as a vehicle for raising awareness of health-related content, and (3) art as a means of self-regulation. These themes were further analyzed using the Relationships and Expectations domain of the PEN-3 (perceptions, enablers, and nurturers) cultural model [31], which includes 3 essential domains: Cultural Identity, Relationships and Expectations, and Cultural Empowerment. Our research explicitly focused on the Relationships and Expectations domain of the PEN-3 model. This involved the following three themes: (1) how the artwork depicts or perceives cervical cancer (perceptions), (2) societal or health system resources that promote cervical cancer prevention (enablers), and (3) the influence of family or relations in decision making in accessing cervical cancer prevention or management (nurturers) [32]. This model has previously been used to deepen understanding of how culture and environment influence health behavior interventions [32].

### Ethical Consideration

This contest was organized as part of the Actions for Collaborative Community Engaged Strategies for HPV (ACCESS-HPV; R01CA271033). Ethical approval was obtained from the Institutional Review Board of the Nigerian Institute of Medical Research (ethics approval number IRB/23/108). Participants were informed about the possibility of using data collected for research purposes. Only participants who consented by selecting “Yes” were included in the study. The collected data were anonymized by removing identifiers such as name and email address and replacing them with a unique ID. Only participants who emerged as winners were compensated.

## Results

### Overview

We received a total of 43 entries (Multimedia Appendix 1). Participants were aged 18-27 (mean 22.2, SD 2.6) years, and all identified as women. The art entries included visual (n=14), audiovisual (n=14), text-based (n=14), and audio (n=1) pieces (Multimedia Appendix 1). Most participants’ entries (42/43, 97.6%) focused on cervical cancer and HPV, including definitions, risk factors, treatment, and prevention. Using the framework from Lakh et al [29] and the Relationships and Expectations domain of the PEN-3 cultural model for thematic analysis, we categorized participants’ entries into 3 emerged themes: perceptions, enablers, and nurturers.

### Art as an Avenue for Mental and Emotional Expression (Perception)

Over two-thirds (27/43, 62.8%) of participants’ entries focused on “perceptions” related to art as a means of emotional expression, encompassing feelings such as humor, sadness, hope, faith, unity, and fear in the context of cervical cancer and its prevention (Table 1). Hope was the most recurring emotion (n=10), as described by one of the participants (entry #01): “Making a difference is not a superpower, and it is not a privilege only to the people who we think can. Sometimes, if we can feel a little bit more and allow our hearts to get stirred, it can lead our feet to where change resides.” The green color was also used to symbolize hope, as explained in the synopsis of entry #08: “I also used the green color to write HOPE because green depicts growth and hope itself, and it is part of our Nigerian color.” Entries also depicted the “fear of death” and “pain” of loss attributed to cervical cancer (entry #04) as a means of emphasizing the need for cervical cancer prevention, “She reminisces about her life before cervical cancer. Then she tells us about the struggles of being a person with cervical cancer patient ...” Other emotions expressed include faith (entry #02), where “women who are metaphorized as “lilies,” “roses,” more than rubies that should take proper care.” Other perceptions reported are those of susceptibility and awareness regarding cervical cancer and its prevention (entry #09), highlighting the crucial role of awareness and early detection and promoting regular screenings and HPV vaccination. One of our entries (#40) also depicted perceptions of unity in the face of diversity to prevent cervical cancer by demonstrating “Nigerian women, who, regardless of age, status, tribe or religion, are susceptible to and share the bane that is cervical cancer.”

**Table 1:** Thematic analysis of the art contest submissions using the Relationships and Expectations domain of the PEN-3 (perceptions, enablers, and nurturers) cultural model.

PEN-3 domain, themes (total entries), and subthemes				Mentions per subthemes, n
**Perceptions**				
	**Art as an outlet for emotional expression**			
		Fear and anxiety	4	
		Humor	1	
		Sadness	1	
		Hope	10	
		Faith	2	
		Others	9	
**Enablers**				
	**Art as a medium for expressing awareness on health-related content**			
		Definition, pathophysiology and epidemiology	7	
		Signs and symptoms, eg, abnormal vaginal bleeding, pelvic discomfort, pain, foul smelling, headache, weight loss, painful urination, diarrhea	10	
		Cause, eg, HPV^a^, unprotected sexual intercourse	5	
		Risk factors	1	
		Treatment, eg, chemotherapy, psychosocial therapy	6	
		Prevention, eg, HPV vaccination, screening, and early detection	34	
**Nurtures**				
	Art expressed as a form of self-regulation			3

^a^HPV: human papillomavirus.

### Art as a Vehicle of Awareness for Health-Related Content (Enabler)

A significant portion (34/43, 79.1%) of entries aimed to promote HPV screening and vaccination. These were classified as “enablers” that highlighted art as a means to foster health awareness, including educational materials and information about cervical cancer. The entries featured infographics, illustrations, poems, drama, explainer videos, and graphics covering definition, epidemiology, signs and symptoms, causes, risk factors, and the treatment and prevention of cervical cancer. Out of the 34 entries, most provided details on preventing cervical cancer, including HPV vaccination, Pap smear tests, HPV screening, and the importance of early detection and management. Common signs and symptoms identified were abnormal vaginal bleeding, pelvic pain, unpleasant odors, weight loss, and headaches. Participants noted that HPV infections might not show symptoms, stating that (entry #16) “most infections with HPV resolve and cause no symptoms.” In addition, artistic entries creatively used symbols to raise awareness about cervical cancer. For example, a syringe represented HPV vaccination (entries #14 and #17), a flower depicted the female reproductive system (entries #20 and #38), and a monster, along with a masked villain, symbolized HPV (entries #21 and #34; Table 1).

### Art as a Form of Self-Regulation (Nurturers)

Finally, entries (3/43, 7%) also included “nurturers” through art, depicted as a form of self-regulation that addresses stress and challenges associated with having relatives with cervical cancer. For instance, entry #15 “portrays the mother’s pains and struggles to protect her daughter and others from the same pain.” The cervical cancer story was also used to encourage people in the community to embrace prevention. The story shared by entry #29 discusses “her personal battle with the disease” and “encourages her community to take proactive steps toward preventing cervical cancer” (Table 1).

### Social Media Promotion and Awareness

The 6 finalists were promoted on social media for 48 hours to enhance awareness of cervical cancer, reaching 8685 accounts and generating 3509 engagements (likes=2727 and comments=782) on Instagram (average likes=454.5). Entry #40, named “Connected in Hope,” attracted the highest engagement (likes=745 and comments=291), whereas entry #43 had the broadest account reach (total=2291), as presented in Table 2. Three winners were determined based on their level of social media engagement and their overall rubric score. The artworks of the 3 winners are provided in reference [26-28].

**Table 2:** Social media engagements and data analytics of the 6 finalist submissions.

Entry number	Title	Likes, n	Comments, n	Total engagement (likes and comment), n	Accounts reached, n
#43	A fighting chance	697	98	795	2291
#19	The Tale of Aunty	432	61	493	692
#41	“it”	125	19	144	1343
#40	Connected in Hope	745	291	1036	1573
#18	Hope	173	78	251	881
#21	“Guardian of the Cervix: The battle against sneaky villain”	555	235	790	1905
Total	—^a^	2727	782	3509	8685

^a^Not applicable.

## Discussion

### Overview

We advance the current literature by illustrating the importance of social media and art in promoting cervical cancer awareness. By using a participatory approach, we demonstrate how engaging art submissions can resonate with audiences, fostering emotional connections and encouraging proactive health measures. This innovative method enhances outreach efforts and empowers women by transforming them into advocates for their health. Such integration of contemporary communication strategies represents a significant contribution to public health discourse, particularly in contexts with limited awareness and resources, such as Nigeria. This study investigated how art can serve as a cultural backdrop for health promotion to eliminate cervical cancer through participatory activities, such as an open call and a social media contest. By using the PEN-3 cultural model, 3 overarching themes emerged.

Most of the entries included themes related to art as a mental and emotional expression regarding cervical cancer and its prevention. Emotion is a physiological experience that occurs in response to an external stimulus and influences an individual’s learning process and behavior [33]. Educational materials that integrate emotional expression can significantly impact individuals and their behaviors [33]. Hope emerged as the most frequent emotional expression in the entries. Hope therapy has proven beneficial in enhancing the quality of life for patients with cancer, though it should be accompanied by evidence-based management and realistic treatment expectations [34]. Research indicates a connection between culture and emotional expression; while certain cultural groups tend to be more expressive regarding their emotions, others may show the opposite behavior [35,36]. Moreover, art has been recognized globally as a means of promoting awareness of cervical cancer. Art as a vehicle of awareness for health-related content (enabler) was expressed in a majority (34/43, 79.1%) of entries, including content promoting HPV screening and vaccination. In India, art illustration was adopted by the Indian Cancer Society to educate the public on cervical cancer [33]. For instance, ancient Kerala Mural arts were used to educate Indian women on self-breast examination [37,38]. Additionally, the illustration of women in the traditional dress of the target population can enhance effective communication and the acceptability of the information.

Art contributes to health education by fostering empathy and creativity, influencing public behavior through cultural context. The submitted artworks reflected societal norms and local languages, enhancing the health message and promoting the intervention’s sustainability. Vickhoff et al [39] noted art’s appeal to cultural values, such as drawing characters in the local dress of the target population. Entry #40 illustrated women wearing attire from the 3 major ethnic groups in Nigeria to promote diversity and inclusion in eliminating cervical cancer. Additionally, entries #20 and #38 used flowers to symbolize the female reproductive system, addressing the cultural stigma around cervical cancer and female sexual health in Nigeria [35,36]. This imagery counteracts the shame associated with cervical cancer, emphasizing the importance of discussing it openly. Entries #21 and #34 depicted HPV as a monster to highlight the virus’s danger and the urgency of prevention. Misconceptions in Africa often link cervical cancer to spiritual beliefs, so these entries symbolize HPV as the “devil” [40,41]. Entry #15 featured the Ibibio word (Ima meaning “love”) in a letter promoting cervical cancer prevention.

Finally, 3 entries used art as a tool for self-regulation and shared experiences to advocate for cervical cancer prevention. A systematic review by Fadlallah et al [42] examined the impact of storytelling on policy advocacy and public awareness. A personal account or story from a cancer survivor or family can attract greater public attention and drive change. Such narratives can positively impact health education, encourage the use of preventive services, and promote engagement for an improved health system that ensures equitable access to services [43]. Using art entries to share the personal experiences of cervical cancer survivors or their loved ones can encourage HPV vaccination among girls and motivate women to undergo regular cervical screenings.

### The Role of Art in Health Empowerment and Communication Through Social Media

Our art contest also showed the potential of art in women’s health empowerment and communication through social media. First, participants expressed creativity through text-based, visual, and audiovisual entries. Although art in public health promotion is relatively new, it has been found to increase engagement compared to other “traditional” methods. Within 48 hours, the 6 finalists reached 8685 social media accounts, generating 2727 likes (455 on average) and 782 comments. This illustrates the potential of social media platforms to enhance the dissemination of educational messages, thus making them more accessible to a broader audience, especially younger women and remote communities.

In contrast, using traditional media for health promotion—such as newspapers, billboards, and television—can be more costly and reach fewer target groups. Moreover, unlike offline media, social media content remains continuously available without extra costs. However, increasing engagement on social media necessitates specific strategies, as not all individuals use social media platforms. A study from Brazil that examined Instagram posts about cervical cancer found an average of 153 likes per post [44]. This variation may stem from the art contest being part of a campaign focused on the cervical elimination day of action. Salako et al [45] noted that social media engagement regarding cancer tends to be greater during global campaigns than before or after those events.

The art contest was open only to females, thereby empowering girls and women to lead change. The art contest not only informed social media users about cervical cancer but also empowered women to act by leading change as health creatives, inspiring girls and women to get screened, vaccinated, or share information within their networks. For instance, entry #01 shared a story of a young woman who initiated community-based awareness and likened this to a “superpower” by saying, “Making a difference is not a superpower, and it is not a privilege only for those we think can.” Entry #06 also centered on empowering girls and women to take charge of their health. One major cultural challenge of cervical cancer vaccination and screening in low- and middle-income countries is the inability of girls and women to make health decisions independently, as men often hold decision-making power. The entry was described as “The girl, a central figure, symbolizes empowerment, and her skin showcases words like ‘Screening’ and ‘Survivor.’” This visual manifesto encourages self-awareness, urges women to take charge of their health, and fosters community solidarity.” Art and social media also helped break down cultural stigmas and misconceptions surrounding cervical cancer and HPV in different communities. Some of the common misconceptions in Nigeria include the association of cervical cancer with promiscuous women, ignorance of risk, and fear of contracting sexually transmitted infections from screening [46].

Additionally, entry #41, titled “it,” used spoken words to creatively lend a voice to the need to discuss cervical cancer publicly without stigmatization, “... what does the silence really do but hurt us? ... all it takes is to break the silence with one person, and maybe our vagina (it) will not worsen.” Lastly, social media promotes peer-to-peer education through the “share” feature. Other social media users shared finalist entries multiple times to drive health conversations on their pages, thereby increasing the reach and impact of the artworks. It also supports the ownership and sustainability of the intervention.

### Implications for Future Public Health Campaigns

The 3 winning art entries [26-28] are currently being used to raise awareness about cervical cancer and vaccination on print and social media. Specifically, the first-place visual was printed and branded on T-shirts and a magazine to raise cancer and vaccination awareness.

As digital engagement continues to expand, leveraging social media will become increasingly vital in increasing the reach of health information, particularly among younger populations. Moreover, incorporating arts and creativity into health communication strategies will be essential for driving more effective health-seeking behaviors. Future initiatives should prioritize people-centered artistic approaches, tailoring content to diverse age groups, cultural backgrounds, and educational levels. By doing so, health messaging can become more inclusive, engaging, and impactful, ultimately fostering a more informed and proactive society in addressing health challenges.

### Study Limitations

Art contests on social media can promote health, but they have some limitations. Although internet access is growing in Nigeria and sub-Saharan Africa, many people, particularly in rural areas, still struggle to access mobile phones and the internet. Older individuals may also be left out of health promotion initiatives because most social media users are younger. Moreover, art interpretation is inherently subjective, influenced by context, culture, and personal perspectives. Consequently, some health messages might not be easily understood without an artist’s explanation.

Additionally, there is potential for bias in the judging of art submissions. Although established judging criteria were designed and 5 independent judges evaluated the entries to minimize bias, the final entries were partly assessed based on social media engagement, which can also be subjective and favors participants with large followings. However, we mitigated this bias by grading the initial submissions solely on quality and by ensuring that the 6 finalists were equally deserving of selection as winners. Nonetheless, this approach enhances the accessibility of art for educating social media users. Lastly, the exclusion of men might have affected the campaign’s reach in educating men to support cervical cancer prevention. This was to adhere to the ACCESS-HPV study protocol. Subsequent art contest or program will explore contributions of men in cervical cancer prevention.

### Conclusion

In conclusion, this study shows the potential of using art and social media to empower women through cervical cancer prevention and screening education. It demonstrates the integration of cultural context in creativity to drive health promotion and increase impact. We encourage health organizations to adopt similar approaches to other women’s health issues, especially those affecting young women, by leveraging creative and culturally relevant tools to increase awareness and promote health equity.

## Data Availability

All data generated or analyzed during this study are not publicly available due to the intellectual property of art owners. Only the winning entries were included with the artists’ approval. Other datasets generated are available from the corresponding author on reasonable request.

## Appendix

Multimedia Appendix 1 Age, type, title, overview, and total scores of the art contest crowdsourcing open call submissions.

## Abbreviations

ACCESS-HPV: Actions for Collaborative Community Engaged Strategies for HPV
HPV: human papillomavirus
PEN-3: perceptions, enablers, and nurturers
SRQR: Standard Reporting for Qualitative Research

## References

[1] Singh D, Vignat J, Lorenzoni V, Eslahi M, Ginsburg O, Lauby-Secretan B, Arbyn M, Basu P, Bray F, Vaccarella S (2023). Global estimates of incidence and mortality of cervical cancer in 2020: a baseline analysis of the WHO Global Cervical Cancer Elimination Initiative. Lancet Glob Health.

[2] (2025). Cervical cancer. World Health Organization.

[3] Lawson O, Ameyan L, Tukur Z, Dunu S, Kerry M, Okuyemi OO, Yusuf Z, Fasawe O, Wiwa O, Hebert KS, Joseph JT, Nwokwu UE, Okpako O, Chime CI (2023). Cervical cancer screening outcomes in public health facilities in three states in Nigeria. BMC Public Health.

[4] (2025). Cancer today. World Health Organization.

[5] Awofeso O, Roberts A, Salako O, Balogun L, Okediji P (2018). Prevalence and pattern of late-stage presentation in women with breast and cervical cancers in Lagos University Teaching Hospital, Nigeria. Niger Med J.

[6] Okunade KS (2020). Human papillomavirus and cervical cancer. J Obstet Gynaecol.

[7] Crosbie EJ, Einstein MH, Franceschi S, Kitchener HC (2013). Human papillomavirus and cervical cancer. Lancet.

[8] Arbyn M, Weiderpass E, Bruni L, de Sanjosé Silvia, Saraiya M, Ferlay J, Bray F (2020). Estimates of incidence and mortality of cervical cancer in 2018: a worldwide analysis. Lancet Glob Health.

[9] Brotherton JM, Bloem PN (2018). Population-based HPV vaccination programmes are safe and effective: 2017 update and the impetus for achieving better global coverage. Best Pract Res Clin Obstet Gynaecol.

[10] (2020). Global strategy to accelerate the elimination of cervical cancer as a public health problem. World Health Organization.

[11] Cervical Cancer Elimination Initiative. World Health Organization.

[12] Olubodun T, Odukoya OO, Balogun MR (2019). Knowledge, attitude and practice of cervical cancer prevention, among women residing in an urban slum in Lagos, South West, Nigeria. Pan Afr Med J.

[13] Ayeni AR, Okesanya OJ, Olaleke NO, Ologun CO, Amisu OB, Lucero-Prisno DE, Ogunwale VO, Abubakar HU, Emery M, Oso TA (2023). Knowledge of cervical cancer, risk factors, and barriers to screening among reproductive women in Nigeria. J Glob Health Sci.

[14] Rimande-Joel R, Ekenedo GO (2019). Knowledge, belief and practice of cervical cancer screening and prevention among women of Taraba, North-East Nigeria. Asian Pac J Cancer Prev.

[15] Cunningham MS, Skrastins E, Fitzpatrick R, Jindal P, Oneko O, Yeates K, Booth CM, Carpenter J, Aronson KJ (2015). Cervical cancer screening and HPV vaccine acceptability among rural and urban women in Kilimanjaro Region, Tanzania. BMJ Open.

[16] Igbokwe C, Ihongo J, Abugu L, Iweama C, Ugbelu J (2024). Influence of cultural beliefs on the utilization of Integrated Maternal, Newborn, and Child Health Services in Benue State, Nigeria. Cureus.

[17] Siette J, Dodds L, Catanzaro M, Allen S (2023). To be or not to be: arts-based approaches in public health messaging for dementia awareness and prevention. Australas J Ageing.

[18] Bunn C, Kalinga C, Mtema O, Abdulla S, Dillip A, Lwanda J, Mtenga SM, Sharp J, Strachan Z, Gray CM, CultureBodies’ Team (2020). Arts-based approaches to promoting health in sub-Saharan Africa: a scoping review. BMJ Glob Health.

[19] World HO, UNICEF (2018). Crowdsourcing in health and health research: a practical guide. World Health Organization.

[20] Baum F, MacDougall C, Smith D (2006). Participatory action research. J Epidemiol Community Health.

[21] Cornish F, Breton N, Moreno-Tabarez U, Delgado J, Rua M, de-Graft Aikins A, Hodgetts D (2023). Participatory action research. Nat Rev Methods Primers.

[22] O'Brien Bridget C, Harris IB, Beckman TJ, Reed DA, Cook DA (2014). Standards for reporting qualitative research: a synthesis of recommendations. Acad Med.

[23] Vaughn L, Jacquez F (2020). Participatory research methods? Choice points in the research process. JPRM.

[24] (2023). Nigeria: leading social media platforms. Statista.

[25] Instagram - an overview. ScienceDirect.

[26] 1st place. Zenodo.

[27] 2nd place winner. Zenodo.

[28] 3rd place winner. Vimeo.

[29] Lakh E, Shamri-Zeevi L, Kalmanowitz D (2021). Art in the time of corona: a thematic analysis. Arts Psychother.

[30] Clarke V, Braun V (2013). Teaching thematic analysis: overcoming challenges and developing strategies for effective learning. The Psychologist.

[31] Airhihenbuwa CO (1989). Perspectives on AIDS in Africa: strategies for prevention and control. AIDS Education and Prevention.

[32] Iwelunmor J, Newsome V, Airhihenbuwa CO (2014). Framing the impact of culture on health: a systematic review of the PEN-3 cultural model and its application in public health research and interventions. Ethn Health.

[33] McConnell MM, Eva KW (2012). The role of emotion in the learning and transfer of clinical skills and knowledge. Acad Med.

[34] Nierop-van Baalen C, Grypdonck M, van Hecke A, Verhaeghe S (2020). Associated factors of hope in cancer patients during treatment: a systematic literature review. J Adv Nurs.

[35] Immordino-Yang MH, Yang X, Damasio H (2016). Cultural modes of expressing emotions influence how emotions are experienced. Emotion.

[36] Tsai JL (2007). Ideal affect: cultural causes and behavioral consequences. Perspect Psychol Sci.

[37] Lake OG-JICCAM Cervical cancer is the 4th most common cancer in women, it is estimated 13,800 women die per year of cervical cancer in the US. Facebook.

[38] admin ARTCAN: Raising Breast Cancer Awareness. Apollo Cancer Centres. Cancer Treatment Centres.

[39] Vickhoff B (2023). Why art? The role of arts in arts and health. Front Psychol.

[40] Hobenu KA, Naab F (2023). A qualitative exploration of the spiritual wellbeing of women with advanced cervical cancer in Ghana. Int J Palliat Nurs.

[41] Solomon K, Tamire M, Solomon N, Bililign N, Kaba M (2023). Misconceptions about female cancers contributing to late presentation to health facilities in Ethiopia: a qualitative study. Int J Womens Health.

[42] Fadlallah R, El-Jardali F, Nomier M, Hemadi N, Arif K, Langlois EV, Akl EA (2019). Using narratives to impact health policy-making: a systematic review. Health Res Policy Syst.

[43] Beck CS, Aubuchon SM, McKenna TP, Ruhl S, Simmons N (2014). Blurring personal health and public priorities: an analysis of celebrity health narratives in the public sphere. Health Commun.

[44] Vicente E, De Faria Sergio Eduardo Emydgio, Almeida AB, Yamada PA, Lucena T, Silva T, Bernuci M (2022). Cervical cancer prevention on Instagram: content and social interaction analysis of Brazilian accounts. Asian Pac J Cancer Prev.

[45] Salako O, Roberts AA, Isibor VI, Babatunde O, Fatiregun O, Nwogu CN (2017). Innovative breast cancer awareness and advocacy campaign. J Glob Oncol.

[46] Mafiana Joy J, Dhital S, Halabia M, Wang X (2022). Barriers to uptake of cervical cancer screening among women in Nigeria: a systematic review. Afr Health Sci.

