# Supplementary material for: Creative Arts to Enhance Cervical Cancer Awareness Using Art-Based Messages From a Nigerian Crowdsourcing Open Call: Qualitative Thematic Analysis
Source: JMIR Form Res. 2026 Jan 23;10:e76240. doi: 10.2196/76240 (PMC12881902; doi:10.2196/76240)
Supplement: Multimedia Appendix 1 [file formative_v10i1e76240_app1.docx]

**Table S1:** Age, type, title, overview, and total scores of the art contest crowdsourcing open call submissions

| **S/N** | **Age (in years)** | **Type (visual, audiovisuals, and text)** | **Title of Submission** | **Synopsis/brief explanation (100 word limit)** | **Total Score** |
| --- | --- | --- | --- | --- | --- |
| #01 | 27 | **Text-based** | A Harmattan Illustration | Making a difference is not a super power and it is not a privilege only to the people who we think can. Sometimes, if we can feel a little bit more and allow our hearts to get stirred, it can lead our feet to where change resides. The illustrator in this work realizes this early and as ordinary as she considers herself, she does extraordinary things...because there’s nothing ordinary about joining the fight against cervical cancer. | **95** |
| #02 | 18 | **Text-based** | Poem | STANZA ONE: Starting with a rhetorical question, the poem illustrates how dear our health is,Thus women who are metaphorized as “lilies” ,”roses” ,more than rubies should take proper care.  STANZA TWO:This stanza Introduce us  to cancer has a deterrent to a flawless health. And spreads even in the cervix Bringing about the term “cervical cancer.” However, its vaccine brings about security.  STANZA THREE:In addressing women and young girls ,the poem deems them fit to be concerned in the vaccination and screening exercise against cervical cancer in order to have “Freedom and Immunity.”  Essentially This poem is an advocate to #Endcervicalcancer. | **76** |
| #03 | 21 | **Visual (infographics)** | In Time | My work is inspired by vintage illustrated magazine adverts. My objective with this illustration was to make an advert of the Human Papilloma Virus vaccine marketed towards young women who I believe are an ideal audience for the promotion of the vaccine due to the strong sense of community associated with this group. With this illustration, I wanted to convey a hopeful outlook towards the elimination of cervical cancer in society as well as present information in an easily digestible way | **88** |
| #04 | 21 | **Text-based** | Poem title: a nemesis for cervical cancer. | This poem talks about a young woman that has been diagnosed with stage IV cervical cancer. She reminisces her life before the cervical cancer. Then she tell us about the struggles of being a cervical patient with cancer. Most importantly, she also decides to spend the remaining time she has creating awareness about HPV vaccine, HPV screenings and tests; As well as cherishing her time with friends and family. | **89** |
| #05 | 24 | **Audiovisual** | Video about cervical cancer awareness. | This video basically sensitize people about the causes, signs and symptoms, the treatment and the HPV vaccine .  It touches all area of cervical cancer as It’s important to sensitize people about the 4th common cancer among women. My video exceeds 10MB so I’m going to send it to the email tagged on this form.  Thank you for your understanding. | **75** |
| #06 | 20 | **Visual** | EmpowHER | “EmpowHER” is a powerful drawing that promotes cervical cancer awareness by imprinting words directly on a girl’s body. This innovative artwork emphasizes empowerment, advocacy, and self-awareness. The girl, a central figure, symbolizes empowerment, and her skin showcases words like “Screening” and “Survivor.” This visual manifesto encourages self-awareness, urges women to take charge of their health, and fosters community solidarity. By wearing these words on her skin, the girl embodies the idea that knowledge is power in the fight against cervical cancer, inspiring individuals to be health advocates and agents of change. | **94** |
| #07 | 22 | **Text-based** | A Poem on Cervical Cancer Elimination | The elimination of cervical cancer refers to the global goal of eradicating this disease as a public health problem. In order to achieve this, various strategies are being implemented worldwide, including widespread access to vaccination against the human papillomavirus (HPV), which is the main cause of cervical cancer. Additionally, regular screening programs, such as Pap smears and HPV testing, are crucial in detecting pre-cancerous lesions early and allowing for timely treatment. Efficient healthcare systems, education and awareness campaigns, as well as improved access to healthcare services, are essential in achieving the elimination of cervical cancer as a significant public health issue. | **88** |
| #08 | 23 | **Visual** | Makeup representation of Cervical Cancer Awareness | Cervical Cancer Awareness is reaching out to women in different age groups on cervical cancer, symptoms, preventive measures and vaccines. Cervical Cancer occurs in the cervix. In my makeup representation, I used red colour to indicate the reproductive system of a woman which consists of the uterus, fallopian tube and the cervix. I used teal colour because that is the colour being used for cervical cancer awareness. I also used the green colour to write HOPE because green depicts growth and hope itself and it is part of our Nigerian colour. Thank you very much. | **100** |
| #09 | 19 | **Text-based** | END CERVICAL CANCER | “End Cervical Cancer” is a poignant poem that advocates the eradication of cervical cancer through the combined power of knowledge and action. It emphasizes the importance of awareness and early detection, encouraging regular screenings and HPV vaccinations. The verses highlight the devastating impact of this preventable disease and the lives it claims. With unity and informed decision-making, the poem calls for a world where cervical cancer is but a memory, emphasizing that through education and proactive measures, we can triumph over this silent killer, sparing countless lives and alleviating the suffering it inflicts. | **83** |
| #10 | 22 | **Audiovisual** | TEAL RIBBON DRESS | The #Endcervicalcancer art challenge is a great way to combine my many interests as a 5th medical student and as a fashion, art and design enthusiast.   The teal and white ribbon represents cervical cancer globally and for the challenge I used these ribbons to create a dress, something inarguably feminine, as we are the ones affected by the cancer.  With this entry, I hope to inform more women about cervical cancer, HPV infection and most importantly vaccination and screening. As a woman, sister and future mother, this is playing my part in reducing the incidence of Cervical cancer in Nigeria. | **103** |
| #11 | 25 | **Audiovisual** | Igniting hope: uniting against cervical cancer | This artwork is a combination of spoken words and visual art. The second image indicates a shadowy figure representing cervical cancer gradually fading as a group of women reach towards a shining light. The third Image illustrates a woman with symptoms of discomfort, pain and bleeding. Next, a woman stands tall ready to win the fight against cervical cancer. Then a scientific laboratory with a beam of light shining on a model of the HPV virus. Then a vaccine guarding women against cervical cancer. Then a key symbolizing prevention within reach of a diverse group of people, emphasizing the importance of education. Women’s hands joined together in unity, reaching out to support and protect each other. Finally, women determined to conquer. | **47** |
| #12 | 25 | **Visual (infographics)** | Engaging Families Through Door-To-Door Outreach For Cervical Cancer Screening and Vaccination | In our community-focused initiative, we plan to implement a comprehensive door-to-door approach to promote cervical cancer screening and vaccination. Recognizing the importance of early detection and prevention, our goal is to reach every household, engage families, and empower them with knowledge about cervical cancer and the available preventive measures. Through this door-to-door initiative, we aspire to create a community that is well-informed, proactive, and supportive of cervical cancer screenings and vaccinations. By engaging families at their doorstep, we believe we can significantly increase awareness, reduce stigma, and contribute to a healthier, empowered community, free from the threat of cervical cancer. | **64** |
| #13 | 19 | **Text-based** | Endcervicalcancer | It a story of young girl who has been neglected by her single mother who was always out in the street hustling to provide. Chineye is taken advantage of on her 14th birthday. For the first time she had sex and it was a great experience for her. She wanted more which she got from other people when John was away in school. She begins to notice changes in her body. She gathers courage to tell her mum. Who took her to a hospital where she ran test. chineye had high risk HPV which has lead to cervical cancer. | **88** |
| #14 | 23 | **Visual** | Vaccinate and support her | Topic: Vaccinate and Support Her Cervical cancer is known to be the most preventable cancer in the world. The common cause of cervical cancer is the Human Papilloma Virus (HPV) and a breach in the cervix during activities like coitus (sexual intercourse) can allow for its penetration, hence, cervical cancer. The theme of my pencil artwork encompasses various outstretched hands, signifying that the girl child needs support in this course. The illustration of an injection being administered shows the need for a vaccination. The schematic diagram of the cervix and a blue ribbon indicates the cervix and cervical cancer awareness respectively. | **72** |
| #15 | 24 | **Text-based** | A Letter to Ima | A Letter to Ima is a poetry piece written as a letter from a mother bedridden with cervical cancer to her daughter, Ima (a 10 year old). It portrays the pains of the mother and her struggle to protect her daughter and others from going through the same pain. In this piece, Ima is vaccinated against HPV, tells her classmates about it and never loses hope of her mother’s survival.  P.S: Ima is an Ibibio word for love. It is also a make of a person. | **101** |
| #16 | 27 | **Visual** | Feminine Fertility | Contemporary art, acrylic on canvas The teal ribbon represents awareness on cervical cancer.   Cervical cancer develops in a woman’s cervix. Almost all cases are linked to infection with high-risk human papillomaviruses (HPV) transmitted through sexual contact. Although most infections with HPV resolve and cause no symptoms, persistent infection can cause cervical cancer in women.    Effective primary (HPV vaccination) and secondary prevention (screening and treating precancerous lesions) will prevent most cervical cancer cases.   When diagnosed, it is one of the most successfully treatable forms of cancer if it is detected early and managed effectively. | **92** |
| #17 | 22 | **visual** | “Pathway to Protection” (acrylic painting, 24/24inches) | “Pathway to Protection” is a painting that symbolizes the journey of HPV vaccination and screening. It portrays the importance of preventive measures in safeguarding individuals from the risks. We can see a woman representing an individual taking control of their health. She takes a confident and determined pose, holding the doctor symbolizing the decision to undergo HPV vaccination and screening, the syringe represents protection.The surrounding colors represent health and growth, while the pathway represents the journey towards HPV prevention and early detection. The lady’s shadow shaped like a microscope emphasizes the importance of regular check-ups and early detection. Written amidst the tree twigs is the statement “HPV screening” | **81** |
| #18 | 24 | **Audiovisual (spoken words)** | Hope | Spoken word is aimed at creating an awareness of cervical cancer and how it can be detected and prevented. Giving hopes to those who have cervical cancer that it is not a death sentence. There is hope for them. | **106** |
| #19 | 23 | **Text-based** | The Tale of Aunty | This poem is a story about someone who was my Aunty in the way Yoruba people call everyone older than them Aunty. She was dear to me even though she was not family and though it is after a decade, it still hurts that her death was avoidable. | **103** |
| #20 | 26 | **Visual** | Nectar | This art piece was painted with food colours.  It is an image of the lower part of a woman with a sunflower on her womb region... I like to remind all women especially cervical patients with cancer that we should always see our body as flowers... it is attractive in all it essence, outwardly and inwardly... nurture and care for your body like a vineyard | **56** |
| #21 | 24 | **Audiovisual** | “Guardian of the Cervix:The battle against sneaky villian” | In “Guardian of the cervix: The battle against sneaky villian,” we embark on an engaging narrative within the realm of women’s health. We encounter the stealthy villain of cervical cancer, often masked as HPV, as it threatens the cervix. Our protagonist faces cryptic warning signs like unusual bleeding and pelvic discomfort. It’s a captivating story of vigilance, protection, and personal heroism on the path to better health. | **113** |
| #22 | 24 | **Visual** | Learn with Bisola about HPV vaccination in school! | Bipolar is a 15 year old girl in SS1 who is learning about HPV vaccines in Biology class. Join her as she learns about how HPV vaccines save the lives of women all over the world! | **95** |
| #23 | 18 | **Text-based** | Poem | My eyes beholdeth, To see the wonders of God, I am enlightened to behold the works of His Hand, How beautiful it is!  My eyes beholdeth, I look to the left and see how wide the earth is, It astonishes me, Am so overwhelmed  My eyes beholdeth, Also,I look to the right to observe nature and see that the Lord is good, His goodness shows His aesthetics and beauty of His work, I tremble at the sight of it.  My eyes beholdeth, Every living and non- living things around me are bright and fearfully made, All Glory to God! | **33** |
| #24 | 18 | **Audiovisual** | End Cervical Cancer | The video is set to enlighten all females about the Cervical Cancer and the possible prevention. A lot of young ladies are unaware of this type of cancer. Every one is familiar with breast cancer and the likes but it’s rare to find information about cervical cancer just anywhere! The goal is to publicize cervical cancer and it’s vaccine. Every one has the right to awareness and the video is set to achieve that. | **85** |
| #25 | 23 | **audiovisual (drama)** | Conquering the Silent Enemy | In a tight-knit family of three, Mama Ijeoma’s life takes a turn when abnormal vaginal bleeding disrupts a family outing. Relying on herbal remedies, her strength continues to wane, tragically leading to her demise. Afterward, the doctor counsels on the cause of death (cervical cancer) and outlines crucial steps for prevention. Ijeoma bravely commits to the Human Papilloma Virus Vaccine regimen and regular Pap smears. This dedication results in a life free from cervical cancer, as she raises her joyful family. This tale highlights the conquerable nature of cervical cancer, but only if prevention is maximized.  Video link: https://drive.google.com/file/d/1SSu0yqm3tj3Vt80rhLH7dBCH7HGtM1bw/view?usp=drivesdk | **113** |
| #26 | 22 | **Visual** | Don’t give up | cervical cancer  Rooted deep inside my luges  A threat to my womanhood causing death amoung the globe A disease that knows me not cervical cancer is destroying my organs  I have seen others suffering from this agony pain was their neighbor An illness that calls for attention daughters beware of cervical cancer beware of HPV  I fear death but pains I couldn’t endure what is the cure? Thanks to NGO who now has the cure to my pains Today we celebrate the elimination of this deadly diseases. | **59** |
| #27 | 23 | **Text-based** | Break The Silence, Protect Against Cervical Cancer. | Cervical cancer is the commonest gynecological cancer in developing countries. It is the 4th commonest cancer among women globally. There were 604,000 new cases and 342,000 deaths in 2020. About 90% occurred in resource-poor settings. It is primarily caused by Human Papilloma Virus (HPV), especially the high-risk types 16 and 18 which account for 70.7% of cases.  Detectable and treatable premalignant lesions precede cervical cancer for years. This poem serves to create awareness on the need for vaccination of young girls aged 9-13 years and routine Pap smear screening in sexually active females for early detection of abnormalities within the cervix. | **89** |
| #28 | 23 | **Audiovisual** | Killer on the loose! | Slow and steady may win the race, but when the life of a woman is the prize, something must be done. Often underestimated, cervical cancer slowly but surely crept it’s way to being the second leading cancer in women globally.  However, we are not unarmed. With HPV vaccinations for girls 9-14 and screening services for women above 30, cervical cancer can and will be eliminated. The world has come together before and can do so again. It takes massive participation that starts with individual proactivity, by men and women alike. Find your role in this, we all have one, we are all needed. | **103** |
| #29 | 19 | **Text-based** | HER STORY | “HER STORY” is a flash fiction piece that follows the journey of a young girl who becomes a beacon of inspiration for her community. In the face of the looming threat of cervical cancer, she courageously shares her personal battle with the disease, highlighting the importance of HPV vaccination and screening. Through her narrative, she not only triumphs over adversity but also encourages her community to take proactive steps towards preventing cervical cancer. This powerful story underscores the significance of early detection and vaccination, fostering awareness and unity in the fight against this life-threatening illness. | **76** |
| #30 | 26 | **Audiovisual (spoken words)** | HPV vaccine (fight against cervical cancer) | Human papilloma virus is the most sexually transmitted infection (STI). HPV spread through skin-to-skin contact. You can get HPV by having virginal, anal or oral sex with someone who has the virus, even if they don’t know signs or symptoms. Most people with HPV do not know they have the infection. They never develop symptoms or health problem from it. Some people find out they have HPV when they get genital warts. Women may find out they have HPV when they get an abnormal pap test(during cervical cancer screening). A vaccine (HPV vaccine)has been introduced to Nigeria and Africa at large to prevent this deadly virus from our girls aged 9 to 26 years before they become sexually active and screening for adult women who are 30 years and above. | **73** |
| #31 | 18 | **Text-based** | A POEM TITLED: GUARDIANS OF WELLNESS | “Guardians of Wellness” is a poetic masterpiece, intertwining art and advocacy to illuminate the crucial mission of HPV vaccination and screening for girls and women. Through lyrical verses, it paints a canvas where health, resilience, and unity converge. The poem is an ode to the courage of those who step into the light of protection and self-care, embracing the shield of vaccination and the gift of early detection. It eloquently celebrates the transformation brought about by art and words, compelling society to embrace the role of guardians of wellness. The poem is a profound call to action, offering a vision of a healthier, empowered future. | **75** |
| #32 | 21 | **Visual** | SCREEN HPV, VACCINATE HPV | Cervical cancer, a malignant tumor of the lowermost part of the uterus, is the fourth on the list of the abnormal growth that affect the female demography. It is caused by the Human Papiloma Virus(HPV).  Wherever the name cervical cancer sounded, it reminds one of cost and losses; the expensive treatment it demands and the lives of beautiful girls and women it is claiming.   Today, armed with screening and vaccination, the female gender now stands a chance against this cancer. With the advent of improved treatment, the threat cervical cancer is now being threatened and on the path of extinction. | **75** |
| #33 | 18 | **Text-based** | Pain, a sign of a bigger problem. | A poem that captures the pain, confusion and other feelings that a woman experiences when she has cervical cancer. | **92** |
| #34 | 22 | **Visual** | The definition of protection and the end of an era | There’s a lot of symbolism in this art piece. The monster’s head representing what HPV does to the cervix, the primarily female warriors, the dull weapons lighting up when held to symbolize the effectiveness of vaccination, even the monster’s hands sign HPV (via sign language). I poured hope into this artwork, praying that this vaccination campaign will bring an end to the suffering of a vast number of girls and women around the country The monster has wrecked a lot of havoc in the past (visualized by the burning city behind it) and condemned too many women to a life of pain and suffering, it’s time to end the destruction! | **92** |
| #35 | 20 | **Visual (infographics)** | Hpv awareness comic | A comic telling a story of an aunt who gets HPV and gets her nieces to get vaccinated | **94** |
| #36 | 20 | **audiovisual** | Letters Mother Left Behind | Sometimes we need someone to remind us to never give up even if it’s almost too late and sometimes not giving up on ourselves is another way of saving someone else because who knows, someone might just be watching. | **87** |
| #37 | 26 | **Text-based** | Guardiance of Our Health | This is a spoken word that gives a narration of how women and girls 4GW have come together to put an end to the silent killer HPV. It goes further to explain the steps to protection which are vaccine and screening. Finally, it talks about the determination to win against HPV | **73** |
| #38 | 25 | **Visual** | The inVASEon | The artwork depicts the female reproductive system symbolically as a neglected flower vase placed on a stool in a garden infested on by a parasitic ‘corpse flower’ with wilted flowers. The purpose of this artwork is to inspire individuals to prioritize the well-being of their reproductive system. It emphasizes the importance of taking preventative measures, such as undergoing HPV vaccination and screening methods for early detection, in order to prevent the development of cervical cancer. Recognizing the significance of timely detection and overcoming hesitancy towards vaccination is crucial, to curtail the spread of cervical cancer to other organs. | **67** |
| #39 | 22 | **audiovisual** | Endcarvicalcancer | Human papillomavirus vaccines are vaccines that prevent infection by certain types of human papillomavirus. Available HPV vaccines protect against either two, four, or nine types of HPV. All HPV vaccines protect against at least HPV types 16 and 18, which cause the greatest risk of cervical cancer. | **81** |
| #40 | 22 | **Visual** | Connected in Hope | The work represents, using Nigerian women, who, regardless of age, status, tribe or religion are susceptible to and share the bane that is cervical cancer. The theme is of ‘unity in the fight against cervical cancer and in the possibilities of finding healing.’ | **109** |
| #41 | 21 | **Audiovisual (spoken words)** | “It” | This piece aims at addressing the issues behind low turn outs on HPV vaccine and calling for action. | **111** |
| #42 | 20 | **audio (poem)** | “HPV Vaccination and Screening.” | HPV is a virus that can cause cancer, but there is a safe and effective vaccine available. The poem encourages listeners to get vaccinated and screened regularly to protect themselves from HPV and its associated cancers. It describes HPV vaccination as a gift that can save lives, and highlights the importance of protecting our children from HPV. The poem concludes by calling for unity and action in the fight against HPV, urging listeners to get vaccinated and screened, and to support efforts to make these preventive measures accessible to everyone. | **97** |
| #43 | 25 | **audiovisual** | A fighting chance | The aim of this work is to promote the HPV vaccine which is crucial in preventing HPV infections and reducing the risk of cervical cancer. To keep spreading awareness and making a difference! | **105** |
